# Spontaneous regression of plasmablastic lymphoma in an elderly human immunodeficiency virus (HIV)-negative patient

**DOI:** 10.1186/s13000-015-0421-y

**Published:** 2015-10-06

**Authors:** Takuro Igawa, Yasuharu Sato, Hotaka Kawai, Eisei Kondo, Mai Takeuchi, Tomoko Miyata-Takata, Katsuyoshi Takata, Tadashi Yoshino

**Affiliations:** Department of Pathology, Okayama University Graduate School of Medicine, Dentistry and Pharmaceutical Sciences, Okayama, Japan; Division of Pathophysiology, Okayama University Graduate School of Health Sciences, 2-5-1 Shikata-cho, Okayama, 700-8558 Japan; Department of Oral Pathology and Medicine, Okayama University Graduate School of Medicine, Dentistry, and Pharmaceutical Sciences, Okayama, Japan; Department of General Medicine, Okayama University Graduate School of Medicine, Dentistry, and Pharmaceutical Sciences, Okayama, Japan

**Keywords:** Plasmablastic lymphoma, Spontaneous regression, Immunosenescence

## Abstract

Plasmablastic lymphoma (PBL) is an aggressive lymphoma commonly associated with human immunodeficiency virus (HIV) infection. Herein we describe a rare case of PBL that spontaneously regressed. An 80-year-old man was referred to our hospital owing to an exophytic gingival tumor in the right maxillary second molar region. He had no significant past medical history, and a screening test for HIV was negative. Imaging showed that the tumor measured 26 × 23 × 16 mm and was confined in the alveolar bone. The tumor was histologically comprised of highly proliferative immunoblastic cells positive for CD138 and Epstein-Barr virus (EBV)-encoded RNA. Monoclonal IgH chain gene rearrangement was detected via polymerase chain reaction. After biopsy and diagnosis of PBL, the tumor began to decrease in size and had apparently disappeared at the time of surgery. There was no histological evidence of a residual lesion in the surgical specimen. In conclusion, a minority of immunosenescence-associated PBLs in the elderly should be recognized as a unique clinicopathological entity distinct from common aggressive PBL.

## Background

Plasmablastic lymphoma (PBL) is a rare subtype of diffuse large B-cell lymphoma (DLBCL), with a median overall survival time of less than one year, initially documented in 1997 [1, 2]. PBL most commonly occurs in the oral cavity of human immunodeficiency virus (HIV)-positive individuals [2]. It is also associated with other immunodeficiency states, such as iatrogenic immunosuppression due to administration of immunosuppressive agents or immunosenescence in elderly adults [2]. Although there seems to be no significant difference in the prognosis of HIV-positive and HIV-negative PBLs [2], rare PBLs in elderly HIV-negative patients without other known immunodeficiency conditions have recently been shown to possess unique clinicopathological features including relatively indolent clinical behavior [3]. It has been proposed that this age-related type of PBL be categorized as PBL of the elderly (PBL-E) [3]. Epstein-Barr virus (EBV) infection has been observed in all cases of PBL-E [3], compared with 50 to 75 % of PBL cases associated with the other immunodeficiency conditions [2].

Spontaneous regression of low-grade lymphoma reportedly occurs in about 10 % of cases [4, 5], whereas spontaneous regression of aggressive lymphoma after biopsy has rarely been observed [6]. Spontaneous regression of DLBCL in patients with rheumatoid arthritis taking methotrexate after immunosuppressant withdrawal has recently been reported [7].

We herein describe a rare case of PBL-E that spontaneously regressed in the absence of any anti-neoplastic treatment.

## Case presentation

An 80-year-old man was referred to our hospital owing to rapid growth of a gingival tumor in the right maxillary second molar region. He had suffered from repeated gingival swelling of this region for 8 months before his visit. Following a diagnosis of apical periodontitis, his right maxillary second molar was extracted 6 weeks before his visit. After an additional mucosal curettage to treat unsuccessful wound healing, the gingiva at the extraction site began to rapidly grow in size. The patient had no significant past medical history including autoimmune diseases and had not taken any immunosuppressive medication.

**Fig. 1:**
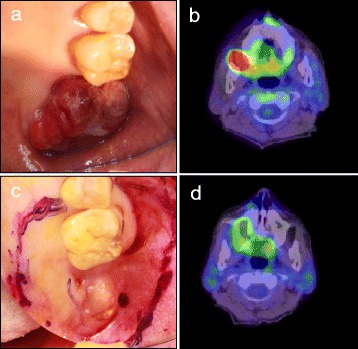
Clinical photographs and imaging data. Clinical photographs (**a**, **c**) and positron emission tomography/computed tomography (PET/CT) imaging (**b**, **d**) of the lesion. Initial presentation (**a**, **b**), 40 days after biopsy when surgery was performed (**c**), and 102 days after biopsy (**d**). The exophytic tumor had clinically disappeared

A physical examination revealed an exophytic gingival tumor in the right maxillary second molar (Fig. 1a). This soft elastic tumor was well circumscribed and bled easily. Computed tomography showed that the tumor measured 26 × 23 × 16 mm and was confined in the alveolar bone. Progression of the tumor to the maxillary antrum was not observed, nor was lymph node swelling. ^18^F-fludeoxyglucose positron emission tomography (FDG-PET) showed elevated FDG uptake in the right maxilla with a maximum standardized uptake value of 29.29 (Fig. 1b). Abnormal FDG uptake at other sites was not noted. Serum levels of lactate dehydrogenase (208 IU/L) and soluble interleukin-2 receptor (177 U/mL) were normal, and a screening test for HIV was negative. Serological tests for EBV were also performed (Table 1).

**Table 1:** Serological tests for EBV and real-time PCR for EBV-DNA in whole blood

Variable	At biopsy	Four days after surgery^a^	Reference (range)
VCA-IgG (titer)	320	80	<10
VCA-IgA (titer)	<10	NA	<10
VCA-IgM (titer)	<10	<10	<10
EA-DR-IgG (titer)	<10	NA	<10
EA-DR-IgA (titer)	<10	NA	<10
EBNA (titer)	20	20	<10
EBV-DNA (copies/μgDNA)	NA	3.7 x 10^2	<1 × 10^2.5

^a^Day 44 after biopsy

*VCA* viral capsid antigen*, EA-DR* early antigen-diffuse and restrict complex, *EBNA* Epstein-Barr virus nuclear antigen, *EBV* Epstein-Barr virus, *NA* not available

**Fig. 2:**
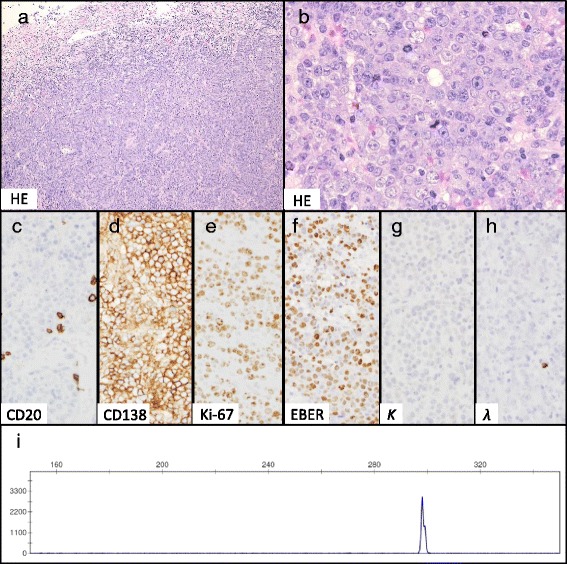
Histology and polymerase chain reaction (PCR) analysis of the lesion at initial presentation. Hematoxylin and eosin staining (**a**, **b**). (**a**) Original magnification, ×100. (b) Original magnification, ×400. Immunohistochemistry for CD20 (**c**), CD138 (**d**), and Ki-67 (**e**) (original magnification, ×400). *In situ* hybridization analyses for Epstein-Barr virus-encoded RNA (EBER) (**f**) and immunoglobulin κ (**g**) and λ (**h**) light chain (original magnification, ×400). PCR analysis for immunoglobulin heavy chain rearrangements (**i**). The lesion was a solid tumor with an ulcertic surface (**a**). Immunoblastic cells with prominent nucleoli (**b**) were negative for CD20 (**c**) but positive for CD138 and EBER (**d**, **f**) with a high Ki-67 index (e). Cytoplasmic immunoglobulin light chain was absent (**e**, **g**). Monoclonal IgH chain gene rearrangement was demonstrated (**i**)

A biopsy of the lesion showed a solid tumor with an ulcertic surface (Fig. 2a). The tumor was characterized by monomorphic neoplastic proliferation of large plasmacytoid and immunoblastic cells with prominent nucleoli (Fig. 2b). Necrosis and giant cells with features similar to those of Hodgkin and Reed/Sternberg cells were not observed. Immunohistochemical immunophenotyping analysis showed that the neoplastic cells were positive for LCA and CD138 and negative for CD20, CD79a, PAX5, CD3, CD5, CD10, CD15, CD56, ALK, LMP1, and EBNA2 (Fig. 2c, d). CD30 expression was not determined. Forty percent of the tumor cells expressed c-Myc, and the Ki-67 labeling index was >80 % (Fig. 2e). As determined via *in situ* hybridization, neoplastic cells were EBV-encoded RNA (EBER)-positive (Fig. 2f). Although cytoplasmic κ and λ light chains were not detected via *in situ* hybridization (Fig. 2g, h), clonal IgH chain gene rearrangement was detected via polymerase chain reaction (PCR) (Fig. 2i). Because the patient had no immunosuppressive condition other than advanced age, he was diagnosed with PBL-E, and surgical excision was scheduled.

**Fig. 3:**
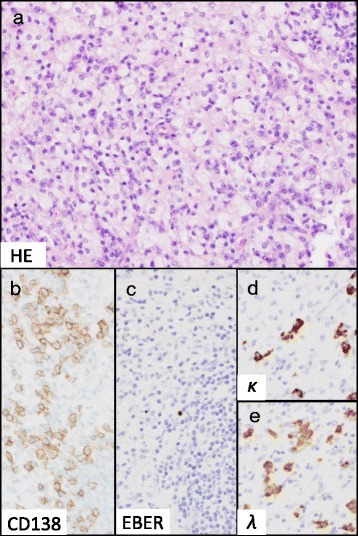
Histology of the surgical specimen. Hematoxylin and eosin staining (original magnification, ×200) (**a**). Immunohistochemical CD138 staining (original magnification, ×400) (**b**). *In situ* hybridization analyses for Epstein-Barr virus-encoded RNA (EBER) (**c**), and immunoglobulin κ (**d**) and λ (**e**) light chain (original magnification, ×400). Plasma cell infiltration was observed along with polymorphic inflammatory cells including numerous foam cells (**a**, **b**). The plasma cells were negative for EBER but expressed cytoplasmic immunoglobulin κ and λ light chain

After the biopsy, however, the tumor began to decrease in size. Surgical excision was performed 40 days after the biopsy, although the exophytic tumor had apparently disappeared (Fig. 1c). A surgical specimen showed infiltration of CD138-positive plasma cells and polymorphic inflammatory cells, including numerous foamy macrophages (Fig. 3a, b). The plasma cells expressed cytoplasmic immunoglobulins (κ and λ light chain) with no light chain restriction, and the results of EBER *in situ* hybridization were negative (Fig. 3c–e). There was no evidence of a residual neoplastic lesion.

Serological testing for EBV was performed 4 days after surgery, and EBV-DNA was detected in whole blood via real-time PCR (Table 1). FDG-PET imaging 102 days after the biopsy showed no abnormal FDG uptake (Fig. 1d), suggesting that the neoplastic lesion had clinically disappeared completely. The patient has thus far been followed-up for 5 months with no sign of relapse.

## Conclusions

PBL is histologically highly aggressive with a high mitotic index [1, 2]. However, the plasmablastic tumor cells in this case completely disappeared in the absence of any anti-tumor treatment after biopsy. A previous report described five cases of age-related EBV-positive mucocutaneous ulcers (EBV-MCUs) that spontaneously regressed without treatment (Table 2) [8]. Interestingly, the PBL-E in our case shares clinical characteristics with these EBV-MCUs, such as old age, mucosa site, a well-circumscribed lesion, ulcer formation, EBV infection, Stage I disease, and a self-limited clinical course [8]. EBV-MCUs are associated with immunosuppressive conditions, such as immunosenescence due to aging, and are considered an indolent EBV-induced lymphoproliferative disorder (LPD) rather than overt lymphomas [8]. Thus far, they have not been associated with HIV infection, and histologically, they contain polymorphous B-cells, including plasmacytoid apoptotic cells and immunoblasts, showing plasmacytic differentiation [8].

**Table 2:** Localized indolent EBV-associated lymphoproliferative disorder/lymphoma in the eldery

No	Age/Sex	Site	Pathologic diagnosis	HIV infection	Treatment	Outcome	Follow-up(months)	IGH/MYC	Reference No. (case No.)
1	80/M	Gingiva	PBL-E	-	None	SR	Alive (8)	N	Present case
2	79/M	Skin of check	EBV-MCU	-	None	SR	DNED (25)	NA	8 (1)
3	82/M	Lip, Skin	EBV-MCU	-	None	SR	NA	NA	8 (2)
4	76/M	Tongue	EBV-MCU	-	None	SR	Alive (12)	NA	8 (7)
5	68/F	Tongue	EBV-MCU	-	None	SR	Alive (36)	NA	8 (13)
6	88/M	Skin of chest	EBV-MCU	-	None	SR	Alive (3)	NA	8 (16)
7	64/M	Nasal cavity	PBL-E	-	CHOP + RT	CR	Alive (55)	N	3 (4)
8	70/M	Gingiva	PBL-E	-	CHOP	CR	Alive (23)	R	3 (6)
9	60/M	Nasal cavity	PBL-E	-	CHOP	Under therapy	Alive (1)	N	3 (8)

*M* male, *F* female, *PBL-E* plasmablastic lymphoma of the elderly, *EBV-MCU* Epstein-Barr virus-positie mucocutaneous ulcer, *HIV* human immunodeficiency virus, *CHOP* cyclophosphamide-adriamycin-vincristine-prednisone, *RT* radiotherapy, *SR* spontaneous regression, CR complete response, *DNED* died no evidence of disease, *NA* not available, *N* negative, *R* rearrangement

Because the PBL-E in our case closely resembles an EBV-MCU, we suggest that it should be considered as an indolent EBV-associated B-cell LPD rather than a common aggressive PBL. It would, however, be considered an atypical EBV-associated LPD owing to its distinctive morphology and immunophenotype. Monomorphically proliferating large lymphoid cells expressing B cell antigens such as CD20 and CD79a are often seen in EBV-associated LPDs [9]. In contrast, the large neoplastic cells observed in our case, which had abundant cytoplasm and prominent nucleoli, expressed CD138 but not CD20 or CD79a. Although necrosis and giant cells resembling Hodgkin and Reed/Sternberg cells are often observed in EBV-associated LPDs, they were not observed in the PBL-E in our case [9].

Similar to our study, a previous report indicated that indolent Stage I PBL-E tumors in three elderly patients had clinical features resembling those of EBV-MCUs (Table 2) [3]. Because these patients received multi-agent chemotherapy soon after diagnosis, it is not known whether their tumors would have regressed spontaneously. To our knowledge, we are the first to report spontaneous regression of a PBL-E. More studies are required to determine the biological features of PBL-E tumors with characteristics similar to those seen in indolent EBV-associated LPDs.

EBV inhibits apoptosis and promotes pathogenesis in EBV-associated LPDs [8]. Although the latency status of EBV in EBV-associated LPDs is usually type II or III, the EBV latency status in our case was type I, in agreement with a previous report of PBL-E [3]. One possible mechanism of the spontaneous regression of the PBL-E is mobilization of the immune system against EBV. In our case, the viral capsid antigen-IgG titer in serum decreased from 1:320 before regression to 1:80 after regression. This change, however, most likely had no significant effect on regression because both titers were within the low range.

*MYC* translocation is a negative prognostic factor for and contributes to the pathogenesis of PBLs [2] including PBL-E [3]. In our case, however, the c-Myc protein was not highly expressed, and *IgH/MYC* translocations were not detected via fluorescence in-situ hybridization. The absence of this translocation may account at least in part for the indolent clinical course of the PBL-E in our case.

In contrast to our case, three previously reported cases of PBL showing spontaneous regression were clearly associated with a specific immunodeficiency (e.g., HIV infection [10, 11] and methotrexate administration [12]). The spontaneous regression in these cases may be related to the patient’s restoration of immune function secondary to anti-HIV treatment or reduced dosage of an immunosuppressive agent. Therefore, the mechanisms underlying the spontaneous regression in our case may differ from those in these previous cases.

In conclusion, PBL-E can partially follow, albeit rarely, a self-limited clinical course without anti-neoplastic therapy. Only a few PBLs associated with immunosenescence have characteristics similar to those of indolent EBV-associated LPDs and should be recognized as a unique clinicopathological entity distinct from common aggressive PBL.

## Consent

Written informed consent was obtained from the patient for publication of this report and any accompanying images.

## Abbreviations

PBL: Plasmablastic lymphoma
DLBCL: Diffuse large B-cell lymphoma
HIV: Human immunodeficiency virus
PBL-E: Plasmablastic lymphoma of the elderly
EBV: Epstein-Barr virus
FDG-PET: ^18^F-fludeoxyglucose positron emission tomography
EBER: Epstein-Barr virus-encoded RNA
PCR: Polymerase chain reaction
EBV-MCU: Epstein-Barr virus-positive mucocutaneous ulcer
LPD: Lymphoproliferative disorder
